# Acute Stress and Need for Psychological Follow‐Up Care in Parents of Children Treated at the Paediatric Intensive Care Unit

**DOI:** 10.1111/nicc.70272

**Published:** 2025-12-02

**Authors:** Emilie Rössler, Katherina Wicklein, Teresa Deffner, Hans Proquitté, Jenny Rosendahl

**Affiliations:** ^1^ Institute of Psychosocial Medicine, Psychotherapy and Psychooncology Jena University Hospital Jena Germany; ^2^ Department of Pediatric and Adolescent Medicine Jena University Hospital Jena Germany; ^3^ Department of Anaesthesiology and Intensive Care Medicine Jena University Hospital Jena Germany

## Abstract

**Background:**

Admission of a child to a paediatric intensive care unit (PICU) is a highly stressful experience for parents and may trigger acute stress disorder (ASD), posing a risk for long‐term post‐traumatic stress disorder (PTSD) and depression. Despite this vulnerability, structured follow‐up care is rarely offered.

**Aim:**

This study examined (1) acute stress, post‐traumatic adjustment and the perceived need for psychological follow‐up care among parents of PICU patients, (2) variables associated with ASD symptoms and the need for follow‐up care, and (3) dyadic associations between mothers' and fathers' distress.

**Study Design:**

An observational study was conducted at the PICU of a University Hospital in Germany between December 2022 and February 2024. Parents of children admitted for ≥ 48 h completed standardised self‐report instruments, including the Acute Stress Disorder Scale (ASDS) and the Post‐traumatic Adjustment Scale (PAS). Logistic regression analyses identified predictors of ASD and the need for follow‐up care; dyadic associations were explored with correlation and intraclass correlation analyses.

**Results:**

Seventy‐seven parents of 52 children participated (58% mothers, median age 38 years). Nearly two‐thirds met diagnostic criteria for ASD, with mothers reporting higher symptom severity. Forty‐two percent screened positive for PTSD risk, and 68% for depression risk. One‐third (34%) expressed a need for psychological follow‐up care, which was strongly associated with PTSD risk (OR = 8.42, 95% CI [2.79–25.38]). Dyadic analyses showed significant correlations of ASD symptoms between mothers and fathers (*r* = 0.42), but not for PTSD or depression risk.

**Conclusions:**

Parents of PICU patients experience high rates of acute stress and are at considerable risk for subsequent psychopathology. The need for psychological support is strongly linked to PTSD risk, and parental distress shows dyadic associations.

**Relevance to Clinical Practice:**

Routine psychosocial screening and structured follow‐up care should be integrated into PICU services. Family‐centred interventions targeting both parents may help mitigate long‐term psychological consequences.

## Impact Statements

- What is known about the topic
  ○ Admission of a child to a PICU is highly stressful for parents and can lead to acute stress, PTSD, anxiety, or depression.
  ○ Psychological follow‐up care for parents after PICU admission is not consistently implemented in clinical practice.
  ○ Parental distress may be interdependent within couples, highlighting the need for family‐centred approaches.
- What this paper adds
  ○ More than half of the parents of PICU patients fulfilled diagnostic criteria for acute stress disorder, and many screened positive for PTSD and depression risk.
  ○ Parents at risk of PTSD were more than eight times as likely to express a need for psychological follow‐up care.
  ○ Dyadic analyses showed significant correlations of acute stress symptoms between mothers and fathers, underscoring the importance of addressing both parents in follow‐up interventions.
  ○ Systematic screening and structured psychosocial services should be integrated into PICU care to identify and support families at the highest risk.

## 1 Introduction

The admission of a child to a paediatric intensive care unit (PICU) is a profoundly stressful event for families, particularly for parents. Common reasons for admission to a paediatric intensive care unit (PICU) include severe infections, traumatic injuries, and postoperative monitoring after major surgery [1]. The PICU environment is characterised by high‐technology interventions, life‐sustaining equipment, and an unpredictable clinical course, all of which can evoke profound emotional strain in families [2]. For parents, this period often involves ongoing uncertainty about their child's survival, exposure to invasive procedures, and disruption of family routines. These factors may trigger acute psychological reactions, with the potential for longer‐term psychiatric consequences if left unaddressed [3, 4, 5]. Mental health issues following the critical illness of a close relative have been encompassed by the term Post‐Intensive Care Syndrome—Family (PICS‐F), which describes new or worsening anxiety, depression, acute stress disorder (ASD), post‐traumatic stress disorder (PTSD), and complicated grief [6]. In paediatric contexts, the subset Post‐Intensive Care Syndrome in paediatrics (PICS‐p) acknowledges the additional challenges faced by parents of critically ill children, including disrupted parental roles, altered family dynamics, and long‐term social and financial repercussions [7].

### 1.1 Acute Stress Disorder in the PICU Context

ASD describes the rapid onset of emotional, cognitive, somatic, and behavioural symptoms following exposure to a traumatic or life‐threatening event [8, 9, 10]. In the ICD‐11, ASD is no longer classified as a mental disorder but as a ‘problem associated with harmful or traumatic events’ [9]. In contrast, the DSM‐5 retains ASD as a formal diagnosis, requiring nine or more symptoms from five categories, including intrusive memories, dissociation, avoidance, and hyperarousal, that develop within 3 days to 4 weeks post‐trauma [10]. In the PICU setting, parental ASD is not uncommon. Prevalence estimates vary depending on methodology and timing, but recent studies indicate that up to 78% of parents report significant acute stress symptoms during their child's PICU stay [11, 12]. Importantly, ASD is a known risk factor for later development of post‐traumatic stress disorder (PTSD) [11, 13].

### 1.2 Post‐Traumatic Stress Disorder and Related Conditions

PTSD is defined in the ICD‐11 as a disorder following exposure to an extremely threatening or horrific event, persisting for weeks to months, and impairing social, occupational, or other important areas of functioning [9]. Symptom clusters include intrusive re‐experiencing, avoidance, and a persistent sense of current threat [14]. In parents of PICU patients, studies have shown a very heterogeneous PTSD prevalence, ranging from 6% to 42% at 3–4 months post‐discharge, from 2% to 57% at 5–6 months, and from 9% to 48% at 8–9 months [11, 12]. Studies with follow‐ups longer than 12 months reported PTSD prevalence rates between 21% and 27% [15]. Additionally, 18% to 62% of parents experience subclinical post‐traumatic stress symptoms. Beyond full PTSD and subclinical symptoms, 23% to 31% of parents report anxiety, and 8% to 17% suffer from depression [15]. Following the intensive care discharge of the child, parental post‐traumatic stress trajectories over the first year are heterogeneous. While resilience or recovering from initial PTSD symptoms is the most common pattern, a significant minority of parents experience persistent or delayed symptoms requiring clinical attention [16, 17].

### 1.3 Risk and Protective Factors

Risk factors for parental psychological morbidity during and after PICU treatment of their child have been grouped into pre‐, peri‐, and post‐traumatic domains [15]. Pre‐traumatic factors include parental psychiatric history, life stress, prior trauma, lower socioeconomic status, and female gender. Peri‐traumatic factors relevant to PICU comprise perceived life‐threat to the child, severity of illness, mechanical ventilation, and prolonged length of stay. Post‐traumatic factors include persistent child morbidity, ongoing medical needs, limited social support, and financial burden [7, 11, 12, 15, 18, 19, 20]. The COVID‐19 pandemic introduced additional peri‐traumatic stressors, with restricted visitation linked to increased intrusive symptoms and hyperarousal in parents [21]. Protective factors are equally important to recognise. Strong family functioning, robust social support networks, empathic communication from healthcare staff, and opportunities for parental involvement in care have been shown to mitigate psychological distress [11].

Understanding parental distress after a child's critical illness requires a dyadic perspective, as the psychological burden of one parent can influence that of the other. Research has shown that PTSD symptoms in mothers and fathers are significantly correlated, and in some dyads, PTSD was diagnosed in both parents [22].

### 1.4 Psychological Support and Follow‐Up Care

Psychological support is an essential part of care within the PICU, addressing the mental health of the critically ill children and their parents [23, 24]. In clinical practice, the provision of structured psychological support for parents during and after PICU admission is inconsistent. Psychological support is not yet a standard component of PICU care in many countries, and when available, it is often limited to part‐time coverage and reactive referral [25, 26, 27].

Interventions evaluated in paediatric settings include intensive care diaries—narrative and photographic records maintained during admission—which have been associated with reduced PTSD symptoms in adult ICU relatives [28, 29, 30] and show promise in PICU contexts [31, 32]. Structured follow‐up clinics or programmes such as Creating Opportunities for Parent Empowerment [33] usually include psychoeducation, parent support after discharge, screening families for the risk of developing psychopathology and offering support to those at high risk, as well as specific interventions to target post‐traumatic stress symptoms [34, 35, 36]. Evidence on the efficacy of these interventions is still sparse, though initial results suggest beneficial effects of follow‐up services to reduce psychopathology after PICU discharge [34, 35].

Nevertheless, such approaches are not yet routinely implemented, and uptake may be limited by infrastructural barriers, parental resistance, and resource constraints [34]. A study based on insurance claims data of 95 070 parents found that only a minority of parents who reported mental health symptoms after PICU treatment of their child were receiving mental healthcare [18].

## 2 Justification for Study

Given the potential downstream effects of parental mental health on family functioning and the child's recovery, it is essential to identify parents at risk for adverse mental health outcomes during and after PICU treatment and to ensure their referral to structured support and follow‐up programmes. A fundamental step toward the implementation of such targeted care is a comprehensive understanding of the parental, child, and treatment‐related factors associated with acute stress disorder symptoms and the risk of adverse post‐traumatic adjustment, in order to inform follow‐up services.

## 3 Aims and Objectives

This study focused on the acute stress experienced by parents during intensive care of their child. The primary aim was to examine acute stress and post‐traumatic adjustment of the parents and to identify risk factors for acute stress, including characteristics related to the parents, the paediatric patient, and the intensive care treatment. Secondary aims were to explore the parents' subjective need for psychological follow‐up care and variables related to this need, as well as to examine dyadic associations of acute stress and adverse post‐traumatic adjustment within parent dyads.

## 4 Design and Methods

### 4.1 Setting and Sample

This observational study was conducted at the PICU of a University Hospital in Germany, which has 10 ICU beds, between December 2022 and February 2024. We included mothers and fathers of children treated at the PICU for at least 48 h who had sufficient German language proficiency. Parents were excluded if the indication for the PICU admission was sedation only or for short‐term diagnostic or interventional measures (preparation for magnetic resonance imaging, or endoscopy). Parents of children receiving palliative care or dying at the PICU were also excluded.

### 4.2 Data Collection Tools and Methods

Parents were asked to complete a questionnaire a few days before their child was discharged from the PICU. The questionnaire comprised questions about demographic characteristics of the participant and the child, standardised measures to assess symptoms of acute stress disorder, post‐traumatic adjustment and the need for psychological follow‐up care. Information about the illness and the treatment of the child was extracted from medical records.

#### 4.2.1 Parent, Child, and Treatment‐Related Variables

The following variables were assessed: age and gender of the parents, having another child, being a single parent (parent‐related variables), age and gender of the paediatric patient, PRISM‐III score, and previous intensive care treatment (child‐related variables), and mechanical ventilation (treatment‐related variable).

#### 4.2.2 Acute Stress Disorder Scale

Symptoms of ASD were assessed using the German version of the Acute Stress Disorder Scale (ASDS) [37], a self‐report version of the Acute Stress Disorder Interview based on the criteria from the DSM‐IV [38, 39]. The ASDS includes 19 items which are rated on a 5‐point Likert scale (1 = not at all; 5 = very much) and assesses the presence of symptoms of re‐experiencing (four items), avoidance (four items), arousal (six items), and dissociation (five items). Responses are summed up, resulting in a total score (range 19–95). A total score of ≥ 56 indicates a high risk of developing PTSD in the further course [38]. A score of ≥ 9 in the dissociation subscale, together with a value of ≥ 28 in all other subscales, corresponds to meeting the diagnostic criteria for an acute stress reaction. In the present study, the internal consistency was high with Cronbach's α = 0.95. The English version of the ASDS exhibits a sensitivity of 95% and a specificity of 83% for a suspected ASD diagnosis, and a sensitivity of 91% and a specificity of 93% for predicting PTSD [38].

#### 4.2.3 Post‐Traumatic Adjustment Scale

We used the German version of the Post‐traumatic Adjustment Scale (PAS) [40] to assess the risk for the development of post‐PICU PTSD or depression. The PAS comprises 10 items assessing pre‐, peri‐, and post‐traumatic factors, each rated on a 5‐point Likert scale. The scale is asymmetrical in its coding: for some items, a score of 0 corresponds to ‘strongly disagree’, whereas for others, 0 corresponds to ‘strongly agree’, reflecting both protective and risk factors for psychopathology. The total score ranges from 0 to 40. A cumulative score of 16 or higher indicates an elevated risk of developing post‐traumatic stress disorder (PTSD), as measured by the Post‐traumatic Adjustment Scale—PTSD subscale (PAS‐P) [41]. In addition to PTSD risk assessment, the tool allows for the identification of individuals at risk for depressive symptomatology. Through a subset of five specific items (items 1, 2, 4, 7, and 8), yielding a depression subscale score (PAS‐D) ranging from 0 to 20. A score of 4 or above on the PAS‐D suggests an increased risk for the development of depression [41]. The psychometric evaluation has demonstrated a sensitivity of 82% and a specificity of 84% of PAS‐P for identifying individuals at risk of PTSD. The PAS‐D, while somewhat lower in diagnostic accuracy, exhibits a sensitivity of 72% and a specificity of 75% for detecting risk of depression [41]. In this study, three items (items 6, 9 and 10) had to be adapted for use with parents in the PICU setting.

### 4.3 Study Size

The sample size calculation is based on a linear regression model with a variance explanation of 26% (*R* ^2^ = 0.26; large effect [42]). A minimum sample size of *N* = 49 is required to achieve a statistical power of 0.80, applying a significance level of *α* = 0.05 and including seven predictors in the model.

### 4.4 Data Analysis

Continuous sociodemographic and clinical characteristics are presented as means and standard deviations for normally distributed data, or as medians together with first (Q1) and third quartile (Q3) values for non‐normally distributed data. Categorical variables are reported as absolute and relative frequencies.

Binary logistic regression analyses were conducted to identify relevant variables associated with ASD diagnosis, and with the subjective need for psychological follow‐up care. Initially, univariate analyses identified variables associated with the respective criterion variable. In a subsequent step, all relevant variables (*p* < 0.2) were included in the regression model [43].

Pearson correlation coefficients and intraclass correlation coefficients (with 95% confidence intervals, CI) were calculated to test for dyadic associations between both parents concerning acute stress and post‐traumatic adjustment scores [44]. IBM SPSS Statistics, version 29.0.0, was used for data analysis.

### 4.5 Ethical and Institutional Approvals

This study was approved by the Ethics Committee of the Friedrich Schiller University Jena (Reg. No. 2022‐2843‐Bef, 12 December 2022) and was conducted in accordance with the ethical principles of the Declaration of Helsinki in its latest version [45].

## 5 Results

### 5.1 Participants

**FIGURE 1:**
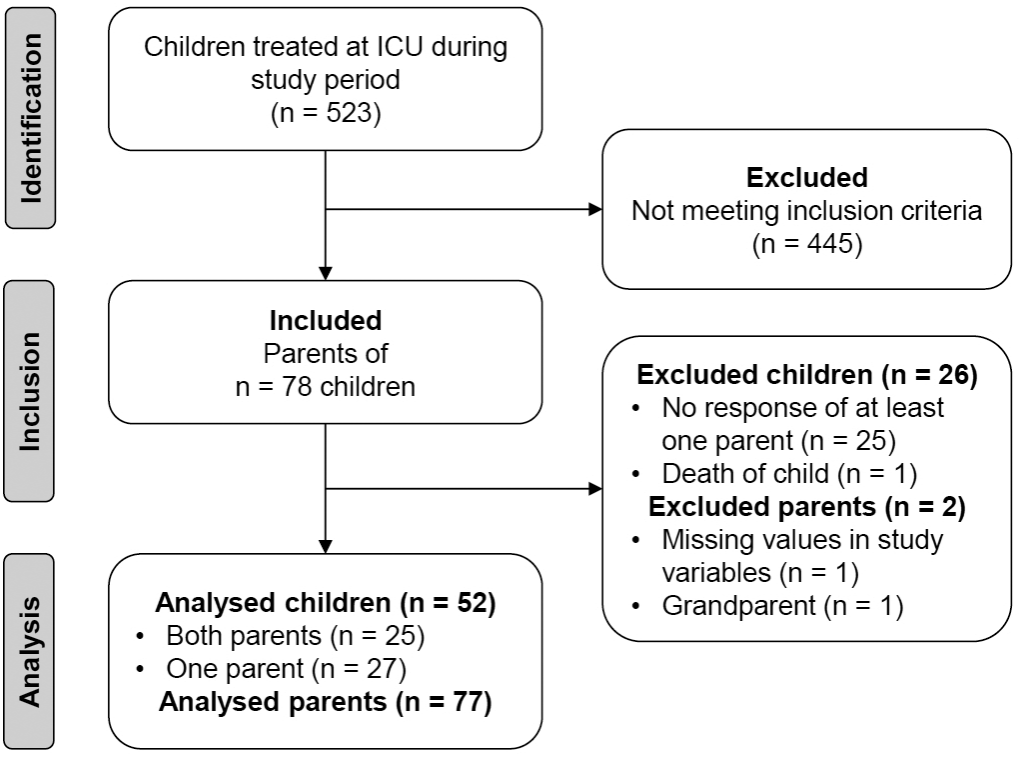
Study flow.

Altogether, 523 children were treated at the PICU during the study period. After screening for eligibility, questionnaires were distributed to the parents of 78 children. Of these, 25 children were excluded because none of their parents returned the questionnaire. One child had to be excluded because he/she died during ICU treatment. After checking for missing values, one parent was excluded due to the large number of missing values in the relevant study variables (PAS‐P, PAS‐D and ASDS). Deviating from the study protocol, we excluded the data of one participating grandparent to maintain homogeneity. However, the data of both related children remained in the analysis because the other parent provided full responses. During data checks, one case with discrepancies between questionnaire data and medical records regarding gender information was detected (both parents indicated female in the questionnaire, while medical records noted female and male). Consequently, this case was excluded from all analyses involving gender as a variable. Finally, the data of 52 children and 77 parents were included in the statistical analyses (Figure 1).

In 25 cases, both parents responded, while for 27 children, we received a response from only one parent. Data were available for 45 mothers (58.4%) and 30 fathers (39%) with a median age of 38 years. The majority of the parents were married (63.6%), and all parents lived with their children in the same household. Six participants (7.8%) were single parents (Table 1). The study included 52 children (24 girls, 28 boys) with a median age of 4 years. A majority of the children (76%) had sibling(s). The median length of ICU stay was 10 days, and the median PRISM‐III score was 5. More than half of the children (53.8%) received mechanical ventilation for a median duration of 2.5 days. Only a minority of the children (17.3%) had a history of previous ICU treatment (Table 1).

**TABLE 1:** Characteristics of the participating parents and their children.

	Parents (*N* = 77)	Children (*N* = 52)
Age, years, median (IQR)^a^	38 (33–42)	4 (< 1; 12)
Gender, *n* (%)^b^		
Female	45 (58.4)	24 (46.2)
Male	30 (39.0)	28 (53.8)
Number of siblings, median (IQR)		1 (0–1)
Parents participating in study, *n* (%)		
Both parents		25 (48.1)
One parent		27 (51.9)
Family status, *n* (%)^c^		
Unmarried	20 (26.0)	
Married	49 (63.6)	
Divorced	4 (5.2)	
Single parenting, *n* (%)^b^		
Yes	6 (7.8)	
No	69 (89.6)	
Length of ICU stay, days, median (IQR)		10 (4–19)
PRISM‐III score, median (IQR)		5 (2–9)
Mechanical ventilation, *n* (%)		
Yes		28 (53.8)
No		24 (46.2)
Length of mechanical ventilation, days; median (IQR)^d^		3 (2–7)
History of previous ICU treatment, *n* (%)		
Yes		9 (17.3)
No		43 (82.7)

Abbreviation: IQR, interquartile range.

^a^ *n* = 1 missing value.

^b^ *n* = 2 missing values.

^c^ *n* = 4 missing values.

^d^ *n* = 28 receiving mechanical ventilation.

### 5.2 Acute Stress, Post‐Traumatic Adjustment, and Subjective Need for Psychological Follow‐Up Care

The mean score of ASDS was 47.85 (SD 14.94), with mothers reporting greater symptom severity than fathers (difference: Cohen's d = 0.33, 95% CI [−0.15; 0.81]; Table 2). Close to two‐thirds of the parents (63.5%) met the criteria for an ASD diagnosis (mothers 66.7%, fathers 53.3%). The median PAS scores were 14 (IQR 10–19) for the PTSD subscale and 5 (IQR 3–8) for the depression subscale. Four out of 10 parents were screened positively for the risk of developing PTSD following PICU treatment of their child (41.6%; mothers: 42.2%, fathers: 40%), and 67.5% of the parents were at risk for subsequent depression (mothers: 73.3%, fathers: 60%). About one‐third of the parents indicated a need for psychological follow‐up care (34.2%; mothers: 39.5%; fathers: 27.6%).

**TABLE 2:** Parental, maternal and paternal scores for the Acute Stress Disorder Scale (ASDS) and the Post‐traumatic Adjustment Scale (PAS).

	All parents *N* = 77^a^	Mothers *N* = 45	Fathers *N* = 30
ASDS total score (range 19–95), M (SD)	47.85 (14.94)^b^	49.7 (14.88)^c^	44.93 (14.84)^d^
ASDS dissociation (range 5–25), M (SD)	12.46 (3.78)^e^	12.61 (3.53)^c^	12.17 (4.23)^c^
ASDS re‐experiencing (range 4–20), Mdn (IQR)	10 (8–13)^f^	11 (7–14)^d^	9 (8–12)^c^
ASDS avoidance (range 4–20), Mdn (IQR)	8 (6–11)^d^	9 (6–11)^c^	9 (6–11)
ASDS hyperarousal (range 6–30), Mdn (IQR)	14 (11–20)^d^	17 (13–20)^c^	13 (11–18)
PAS PTSD score (range 0–40), M (SD)	14.93 (5.75)^e^	15.41 (5.7)^c^	14.28 (5.96)^c^
PAS depression score (range 0–20), Mdn (IQR)	5 (3–8)	6 (3–9)	5 (3–7)

Abbreviation: IQR, interquartile range.

^a^ Including *n* = 2 with a missing value for gender.

^b^ *n* = 5 missing values.

^c^ *n* = 1 missing value.

^d^ *n* = 2 missing values.

^e^ *n* = 3 missing values.

^f^ *n* = 4 missing values.

Parental age, the child's age, and previous PICU treatment were associated with ASD diagnosis in univariate analyses (*p* < 0.2; Table S1). In a multiple binary logistic regression model, none of these variables was significantly associated with the parental ASD diagnosis.

The following variables were associated with subjective need for psychological follow‐up care (*p* < 0.2 in univariate analyses; Table S2) and were included in the multiple binary logistic regression model: ASD diagnosis, post‐traumatic adjustment (risk for PTSD and risk for depression according to PAS), child's age, and previous PICU treatment. In the multiple regression model, the only significant variable associated with the need for follow‐up care was the risk for PTSD (according to PAS‐P, *p* < 0.001). Parents with poor post‐traumatic adjustment and thus at risk for subsequent PTSD reported a subjective need for psychological follow‐up care eight times more often than parents without such a risk (OR = 8.42, 95% CI [2.79; 25.38]). Based on the model, 75% of the parents could be correctly classified. Nagelkerke's *R* ^2^ was 0.28.

### 5.3 Dyadic Parental Associations

**FIGURE 2:**
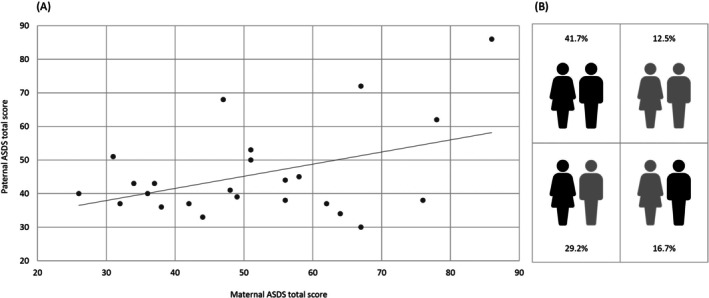
Dyadic association between maternal and paternal ASDS total scores (A) and ASD diagnosis (B). Black shaded icons = ASD diagnosis, grey shaded icons = no ASD diagnosis.

For 25 children, both parents responded, and *n* = 24 provided complete data for inclusion in the following dyadic data analysis (*n* = 23 for the PAS‐P analysis). The severity of acute stress disorder symptoms was significantly correlated between mothers and fathers (*r* = 0.422, 95% CI [0.023; 0.706], *p* = 0.040, *R* ^2^ = 0.18; Figure 2A). Similarly, the intraclass correlation was significant (ICC = 0.568, 95% CI [0.052; 0.809], *p* = 0.019). In 10 parental dyads (41.7%), both, the mother and father, were identified with an ASD diagnosis, while in seven dyads (29.2%) only the mother, and in four dyads (16.7%) only the father exhibited symptoms meeting the ASD diagnostic criteria (Figure 2B).

Dyadic associations were not significant, either for PAS PTSD scores (*r* = 0.134 [−0.295; 0.517], *p* = 0.543, *R* ^2^ = 0.018; ICC = 0.291 [−0.676; 0.696]) or for PAS depression scores (*r* = 0.038 [−0.371; 0.435], *p* = 0.86, *R* ^2^ = 0.007; ICC = 0.083 [−1183; 0.604]).

In five parental dyads (21.7%), both, the mother and the father, were screened for high PTSD risk, while in seven dyads (30.4%) only the mother, and in three dyads (13%) only the father exhibited a high PTSD risk. In 11 parental dyads (45.8%), both, mother and father, were screened for high depression risk, while in six dyads (25%), only the mother, and in three dyads (12.5%), only the father had a high risk for developing subsequent depression.

In four dyads (16.7%), both parents expressed a subjective need for psychological follow‐up care. Mothers, but not fathers, reported such a need in six dyads (25%), and fathers only in two dyads (8.3%). In 11 dyads of parents, neither the mother nor the father declared a need for psychological follow‐up care.

## 6 Discussion

### 6.1 Key Results and Interpretation

This study examined acute stress and post‐traumatic adjustment in parents of children admitted to a PICU, as well as their perceived need for psychological follow‐up care. More than half of the participating parents met diagnostic criteria for ASD, and a substantial proportion screened positively for risk of PTSD and depression. Importantly, the need for psychological follow‐up care perceived by the parents was associated with poor post‐traumatic adjustment in terms of an increased risk for PTSD. Parents at risk for PTSD were more than eight times as likely to express such a need, and the logistic regression model correctly classified 75% of cases (Nagelkerke's *R* ^2^ = 0.28). Dyadic analyses revealed significant associations of ASD symptoms between mothers and fathers, underscoring the interdependence of parental distress. Together, these findings highlight both the high burden of acute stress in this vulnerable population and the importance of systematically identifying families in need of psychological support.

### 6.2 Comparison With Previous Research

Our findings are consistent with earlier studies reporting high rates of acute stress and subsequent psychiatric morbidity in parents during and after PICU admissions [11, 12, 13]. The observed prevalence of ASD symptoms (63.5%) falls within the upper range of prior estimates, likely reflecting the methodological differences in the timing of assessment and diagnostic criteria. In line with previous evidence, mothers tended to report greater symptom severity than fathers, though differences were modest [16]. The strong predictive value of PTSD risk for perceived need of follow‐up care extends earlier work showing that parents often recognise their psychological vulnerability [18]. Dyadic correlations confirm the relevance of a family‐centred perspective, echoing studies showing interdependence of parental distress within couples [22].

### 6.3 Generalisability

The study was conducted in a tertiary PICU in Germany, which may limit transferability to settings with different healthcare systems, psychosocial support infrastructures, or cultural expectations regarding parental involvement. However, the prevalence of acute stress and its association with the need for psychological follow‐up is likely to be relevant across PICU contexts internationally, particularly given converging evidence from recent meta‐analyses [11, 12].

## 7 Strengths and Limitations

Strengths of this study include the prospective observational design, validated assessment instruments with high internal consistency, and the inclusion of both parents whenever possible. The focus on both acute stress and perceived need for care adds clinical relevance, as it informs potential pathways for intervention. Nevertheless, several limitations must be acknowledged. First, the single‐centre setting and modest sample size may limit statistical power and generalisability. Second, the cross‐sectional design precludes causal inferences. Since no follow‐up assessments were conducted, it remains unclear whether parents screened at risk actually went on to develop PTSD or depression. Third, we did not consider all variables in our study that have been identified as risk factors for parental acute stress during PICU treatment of the child, for example, socioeconomic status, prior psychiatric history, prior trauma, and life stress of the parents. Fourth, while self‐report measures capture parents' perspectives, they may be subject to recall or social desirability bias. Finally, exclusion of bereaved parents restricts insights into a group likely at an even higher risk of psychopathology.

## 8 Recommendations and Implications for Practice and Research

Our results underscore the need for systematic psychological screening of parents during and after PICU admissions. Identifying parents at risk for PTSD or depression and proactively offering follow‐up services should be integrated into routine care. Multidisciplinary models embedding mental health professionals in PICUs, as piloted in several countries [25, 27], may serve as feasible approaches. Structured interventions such as follow‐up clinics, parent empowerment programmes, and PICU diaries should be further evaluated and, if effective, scaled up in practice. Future studies should build on our findings by incorporating longitudinal designs with follow‐up assessments to determine whether parents identified as at risk subsequently develop PTSD or depression. Research should also focus on the evaluation of the effectiveness of early psychosocial interventions in preventing post‐PICU mental health impairments, and on the exploration of barriers to the use of follow‐up services. Including dyadic or systemic research approaches could help to better understand the dynamics within the family system.

## 9 Conclusion

In conclusion, the majority of parents of children admitted to a PICU experience clinically relevant acute stress symptoms, with a large proportion at risk for subsequent PTSD and depression. Perceived need for psychological follow‐up care is strongly linked to PTSD risk, and parental distress shows dyadic associations. These findings emphasise the importance of systematic psychosocial assessment and the implementation of structured follow‐up services to support families after paediatric critical illness.

## Funding

The authors have nothing to report.

## Ethics Statement

The study was approved by the ethics committee of the Friedrich‐Schiller University Jena, Germany (number 2022‐2843‐Bef, 12 December 2022).

## Consent

All eligible participants were informed orally by the ER about the aims, content, and procedure of the study. After providing the opportunity to ask questions, written consent was obtained from the participants.

## Conflicts of Interest

The authors declare no conflicts of interest.

## Supporting information


**Table S1:** Univariate associations with ASD diagnosis.
**Table S2:** Univariate associations with need for psychological follow‐up care.

## Data Availability

The data that support the findings of this study are available on request from the corresponding author. The data are not publicly available due to privacy or ethical restrictions.
