# Supplementary material for: Acute Stress and Need for Psychological Follow‐Up Care in Parents of Children Treated at the Paediatric Intensive Care Unit
Source: Nurs Crit Care. 2025 Dec 2;31(1):e70272. doi: 10.1111/nicc.70272 (PMC12672213; doi:10.1111/nicc.70272)
Supplement: Supplementary file 1 — Table S1: Univariate associations with ASD diagnosis. Table S2: Univariate associations with need for psychological follow‐up care. [file NICC-31-0-s001.docx]

**Acute stress and need for psychological follow-up care in parents of children treated at the pediatric intensive care unit**

**Supplementary material**

Table S1. Univariate associations with ASD diagnosis

|  | OR | 95% CI | p |
| --- | --- | --- | --- |
| **Parental age** | **1.07** | **0.99; 1.16** | **0.110** |
| Parental gender^a^ | 0.53 | 0.2; 1.42 | 0.208 |
| > 1 child | 0.91 | 0.31; 2.67 | 0.859 |
| **Age of child** | **1.08** | **0.99; 1.19** | **0.089** |
| PRISM-III score | 0.96 | 0.84; 1.09 | 0.497 |
| **Previous PICU treatment** | **0.43** | **0.12; 1.56** | **0.199** |
| Mechanical ventilation | 1.53 | 0.59; 3.98 | 0.383 |

^a^ female = 0, male = 1

Table S2. Univariate associations with need for psychological follow-up care

|  | OR | 95% CI | p |
| --- | --- | --- | --- |
| **ASD diagnosis** | **7.65** | **2.01; 29.08** | **0.003** |
| **Risk for PTSD (PAS-P)** | **8.88** | **2.95; 26.72** | **< 0.001** |
| **Risk for depression (PAS-D)** | **5.7** | **1.5; 21.66** | **0.011** |
| Parental age | 1.01 | 0.93; 1.09 | 0.989 |
| Parental gender^a^ | 0.56 | 0.2; 1.53 | 0.257 |
| > 1 child | 1.61 | 0.5; 5.18 | 0.422 |
| **Age of child** | **1.06** | **0.97; 1.15** | **0.180** |
| PRISM-III score | 1.03 | 0.91; 1.16 | 0.643 |
| **Previous PICU treatment** | **0.14** | **0.02; 1.19** | **0.072** |
| **Mechanical ventilation** | **2.4** | **0.88; 6.6** | **0.089** |

^a^ female = 0, male = 1
